# Prostate-specific membrane antigen-based imaging for stereotactic irradiation of low-volume progressive prostate cancer: a single-center experience

**DOI:** 10.3389/fonc.2023.1166665

**Published:** 2023-08-10

**Authors:** Linda Varga, Zsuzsanna Besenyi, Viktor R. Paczona, István Farkas, Szabolcs Urbán, Gábor Sipka, László Pávics, Zoltan Varga, Emese Fodor, Katalin Hideghéty, Judit Olah, Zoltán Bajory, Anikó Maráz

**Affiliations:** ^1^ Department of Oncotherapy, University of Szeged, Szeged, Hungary; ^2^ Department of Nuclear Medicine, University of Szeged, Szeged, Hungary; ^3^ Department of Urology, University of Szeged, Szeged, Hungary

**Keywords:** recurrent/oligometastatic prostate cancer, radiation therapy, stereotactic irradiation, PSMA-PET/CT, PSMA-SPECT/CT

## Abstract

**Introduction:**

Prostate-specific membrane antigen (PSMA) is a transmembrane protein that may be expressed on the surface of prostate cancer (PC) cells. It enables a more sensitive and specific diagnosis PC, compared to conventional anatomical imaging.

**Aim:**

The integration of PSMA-based imaging in the personalized radiotherapy of PC patients and the evaluation of its impact on target volume definition if stereotactic body radiotherapy (SBRT) is planned for locally recurrent or oligometastatic disease.

**Patients and methods:**

The data from 363 examinations were analyzed retrospectively. Inclusion criteria were histologically verified PC and clinical data suggesting local recurrence or distant metastasis. Whole-body ^99m^Tc-PSMA-I&S single-photon emission computed tomography (SPECT)/CT or ^18^F-JK-PSMA-7 positron emission tomography/computer tomography (PET/CT) was carried out, and the evaluation of the scans and biological tumor volume contouring was performed at the Department of Nuclear Medicine. The target volume delineation on topometric CT (TCT) scan was performed at the Department of Oncotherapy. The comparison of the two volumes was performed by image fusion and registration.

**Results:**

From 363 PSMA isotope-based examinations, 84 lesions of 64 patients were treated with SBRT. In 50 patients, 70 lesions were examined for intermodality comparison. The target volume defined by the PSMA density was significantly smaller than the tumor size defined by the TCT scan: GTV_CT_ (gross tumor volume on the TCT), 27.58 ± 46.07 cm^3^; BTV_PSMA_ (biological target volume on the PSMA-based examination), 16.14 ± 29.87 cm^3^. During geometrical analyses, the Dice similarity coefficient (DSC) was 0.56 ± 0.20 (0.07–0.85). Prostate-specific antigen (PSA) control was performed to evaluate the response: mean pre-radiotherapy (pre-RT) PSA was 16.98 ng/ml ( ± SD: 33.81), and post-RT PSA at 3 months after SBRT was 11.19 ng/ml ( ± SD: 32.85). Three-month post-therapy PSMA-based imaging was performed in 14 cases, in which we observed a decrease or cessation of isotope uptake. Conventional imaging control was performed in 42 cases (65.6% of all cases): 22 (52.4%) complete remissions, 14 (33.3%) partial remissions, four (9.5%) stable diseases, and two (4.8%) progressive diseases were described.

**Conclusion:**

PSMA-based imaging is a promising diagnostic method for specifying the stage and detecting the low-volume progression. Our results suggest that PSMA-based hybrid imaging can influence treatment decisions and target volume delineation for SBRT.

## Introduction

Prostate cancer is the second most common malignancy among men worldwide (1). Its treatment has vastly improved due to adjuvant androgen deprivation treatment and the increased efficacy of radiotherapy (RT) (2). Recently, as a consequence of the new isotopic modalities, the accuracy of diagnosis and treatment has continued to improve.

An increasing number of studies prove that prostate-specific membrane antigen (PSMA)-based positron emission tomography/computer tomography (PET/CT) provides excellent accuracy and can be a suitable replacement for conventional imaging methods (CT and bone scintigraphy) in primary staging, as well as in restaging of patients with biochemical recurrence. PSMA-based molecular imaging is emerging as the most promising tool in this field (3).

There is growing interest in using PSMA-based imaging in gross target volume delineation for radiation therapy planning since molecular imaging enables the delineation of the biologically active tumor—based on increased PSMA expression on specific information—essential for effective treatment. It also allows the potential identification of small lymph nodes and small bone or hidden soft tissue metastases, missed by conventional CT and magnetic resonance imaging (MRI). Nevertheless, PSMA PET/CT is still under evaluation for the RT target definition (4).

Although PSMA-PET tracers have extensively been investigated and an increasing number of studies prove the efficacy of PSMA-PET, fewer data exist on investigations with PSMA–single-photon emission computed tomography (SPECT) radiopharmaceuticals, but several studies have demonstrated that ^99m^Tc-PSMA-SPECT/CT could be a reasonable and cost-effective alternative to PSMA-PET/CT (5–8).

The basic principles of PSMA imaging are the same. The same target molecule (PSMA) is labeled with ^99m^Tc for SPECT and with positron-emitting isotopes (^68^Ga and ^18^F) for PET imaging. Furthermore, to the best of our knowledge, this is the first study to investigate the role of ^99m^Tc-PSMA SPECT/CT for radiotherapy planning.

In an earlier pilot study, we analyzed data from 19 patients. In 52.6% (10 cases), the PSMA-SPECT/CT revealed a more advanced disease than conventional imaging modalities, and in 15.8% (three cases), osseal and/or lymphatic metastases suggested on CT examination could be excluded. The target volume was unaffected by the PSMA-SPECT/CT findings in only 31.6% (six cases). Definitive radiotherapy was carried out in 15 cases (78.9%). In four cases (21.1%), we had to opt for systemic treatment due to disseminated disease. If only conventional imaging techniques were used to define the target volume, the volume would be on average 2.2 times larger (1.3–4.6) than the one based on PSMA-SPECT/CT (9).

In the last 5 years, the number of prostate cancer patients treated at our Institute is steadily increasing. In 2021 and 2022, 210 and 201 new subjects with histologically verified prostate adenocarcinoma were taken into care, respectively.

## Aim

Our aim was the integration of PSMA-based imaging in the personalized process of radiation therapy of prostate cancer patients and the evaluation of its impact on target volume definition, especially for stereotactic body radiotherapy (SBRT) in the case of postoperative local recurrence and oligometastatic prostate tumors.

The study was approved by the Regional Committee for Human Medical Research Council (229/2017-SZTE).

## Materials

PSMA-based examinations were carried out between 14 November 2017 and 4 October 2022, from which patients who received definitive radiotherapy at the Department of Oncotherapy, University of Szeged, were selected for our present study. PSMA-positive lesions were irradiated using a stereotaxic technique, the delivered dose was prescribed according to international guidelines, and the dose per fraction was always higher than 5 Gy (10).

Inclusion criteria were histologically verified prostatic adenocarcinoma and clinical data (prostate-specific antigen (PSA) level and conventional imaging) suggesting local recurrence after radical prostatectomy (RP) or distant metastasis. If the clinical data, such as PSA level and conventional imaging findings (chest–abdomen–pelvis CT scan, bone scintigraphy) suggested that residual, recurrent, or metastatic tumor tissue might be present, PSMA-based nuclear imaging was carried out. The findings were later used in the target volume definition.

Patients with multiple metastases and cases of palliative radiotherapy were excluded from this study. Those who were not suitable for stereotaxic radiotherapy (performance status) or did not accept the treatment were also left out.

## Methods

### Imaging

In 2017, we started our work with SPECT-CT, and in 2022, we switched to PET/CT.

#### PSMA PET/CT

The PET/CT scans were performed on a GE Discovery IQ Gen 2 PET/CT System (GE Healthcare, Chicago, IL, USA), and the acquisitions were performed 90 min post-injection of 3.7 MBq/kg of intravenous [^18^F]fluoro-JK-PSMA ((2*S*)-2-({{(1*S*)-1-carboxy-5-[(6-[^18^F]fluoro-2-methoxypyridin-3-yl)formamido]pentyl}carbamoil}amino)pentanedioicacid). The PET scan was performed in three-dimensional (3D) modes for 2.5 min per bed position, the field of view was 20 cm with 30% overlap, and the data collection was completed by plain low-dose CT (120 kV and 70 mA·s) mapping). The routine whole-body mapping was performed extending from the skull base to the upper third of the thighs.

#### PSMA SPECT/CT

**Figure 1:**
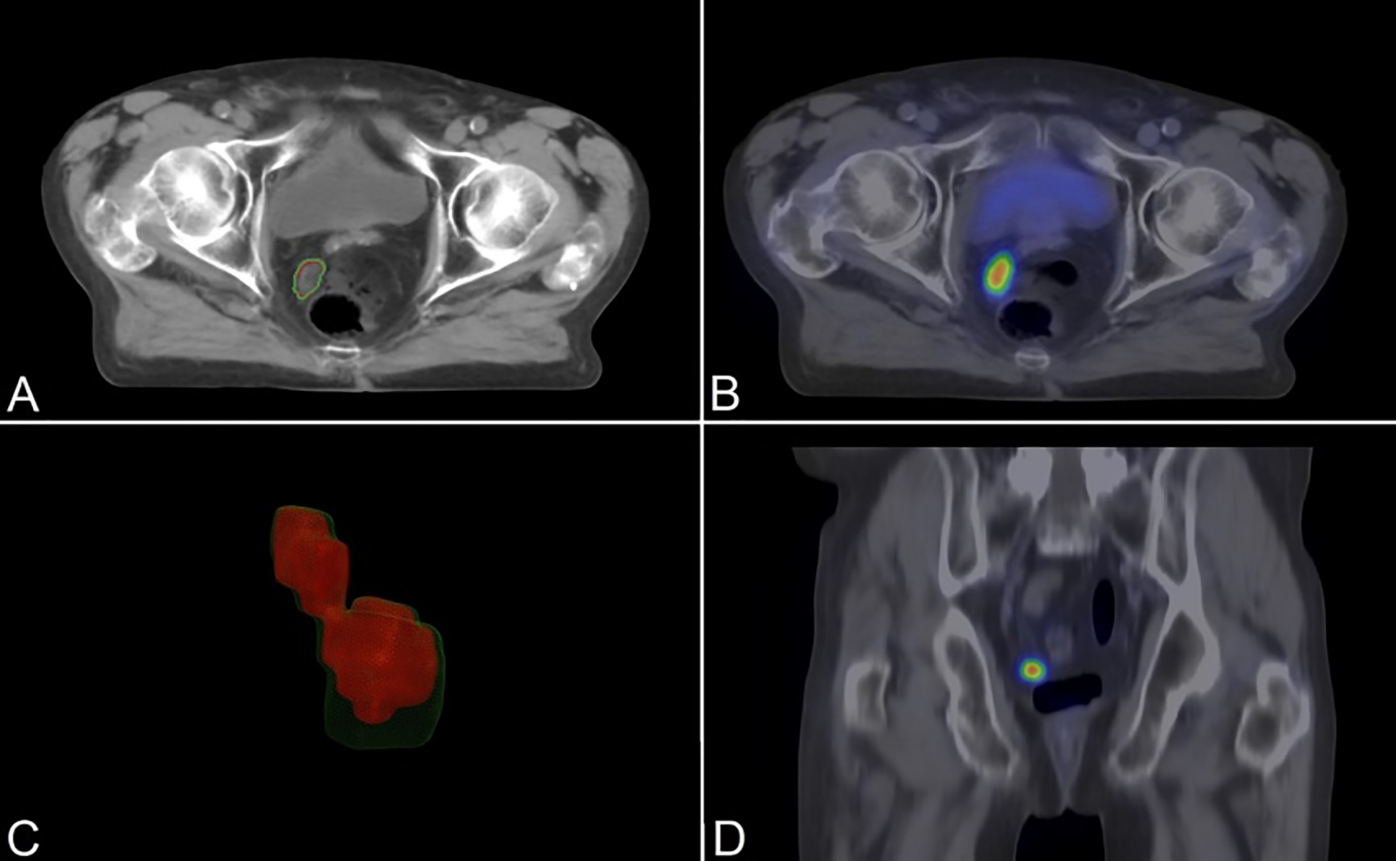
An 81-year-old patient with PSMA-positive enlarged pelvic lymph node (PSA 6.20 ng/ml; Gleason: 3 + 4 = 7). Topometric CT (axial slice **(A)**) fused and registered with ^18^F-PSMA-PET images (axial **(B)** and coronal slices **(D)**). Smoothed 3D polygon model of the GTV_CT_ (green outlines) and BTV_PSMA_ (red outlines) **(C)**. PSMA, prostate-specific membrane antigen; PSA, prostate-specific antigen; GTV_CT_, gross tumor volume on the topometric CT; BTV_PSMA_, biological target volume on the PSMA-based examination.

**Figure 2:**
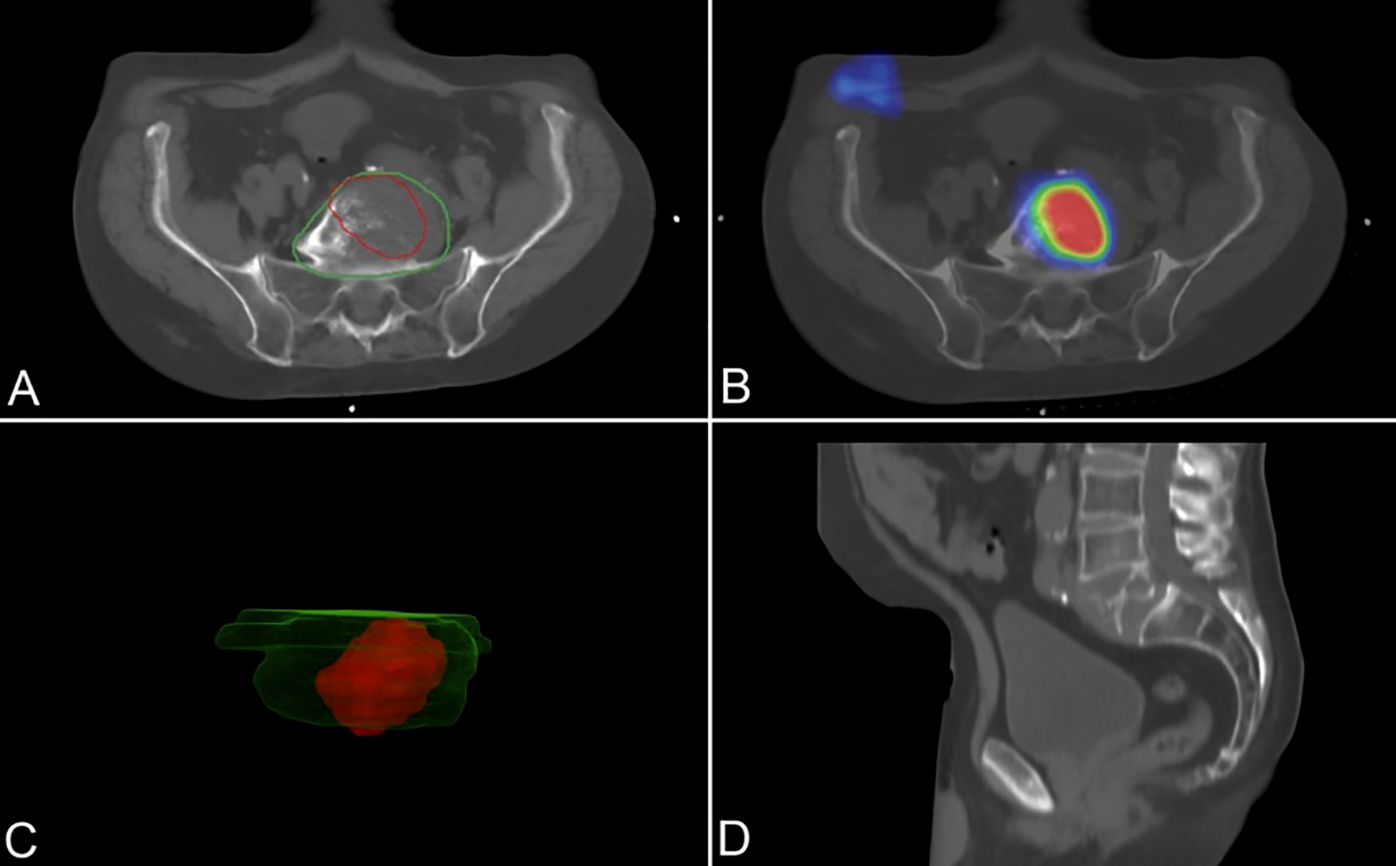
A 74-year-old patient with PSMA-positive lytic vertebral metastasis (PSA 90.68 ng/ml; Gleason: 3 + 4 = 7). Topometric CT (axial **(A)** and sagittal **(D)** slices) fused and registered with ^99m^Tc-PSMA-SPECT images (axial slice **(B)**). Smoothed 3D polygon model of the GTV_CT_ (green outlines) and BTV_PSMA_ (red outlines) **(C)**. PSMA, prostate-specific membrane antigen; PSA, prostate-specific antigen; SPECT, single-photon emission computed tomography; GTV_CT_, gross tumor volume on the topometric CT; BTV_PSMA_, biological target volume on the PSMA-based examination.

Mean activity of 668 ± 95 MBq ^99m^Tc-mas3-y-nal-k(Sub-KuE) (Institute of Isotopes Co. Ltd., Budapest, Hungary) was administered intravenously. Prior to imaging, patients were given oral contrast material (1,000 ml of polyethylene glycol solution) to drink continuously, starting 60 min before the examination. Scans were performed on an integrated whole-body SPECT/CT system (Mediso AnyscanTRIO, Mediso Medical Imaging Systems Ltd., Budapest, Hungary). The whole-body SPECT imaging was carried out 6 h after the administration of the radiopharmaceutical (360°; 96 projections, 10 s/frame, matrix 128 × 128, reconstructed pixel size 4.22 mm). SPECT data collection was completed by low-dose CT (120 kV and 70 mA·s) acquisition (**Figures 1**, **2**).

#### Treatment planning CT

During patient preparation, the target region was positioned and immobilized with All-in-One (AIO) Solution (ORFIT, Wijnegem, Belgium), with individual immobilization system and six-point thermoplastic mask fixation (Pelvicast system, ORFIT, Wijnegem, Belgium) depending on the target area. Treatment planning CT (topometric CT (TCT)) was carried out according to institution protocols and was performed on a Somatom Emotion 6 CT simulator (Siemens, Erlangen, Germany) with CT slices being acquired every 3 mm. Target volumes and organs at risk were delineated after image fusion in the ARIA Oncology Information System (Varian Oncology Systems, Palo Alto, CA, USA) with the review of an experienced radiologist in all cases, based on the recommendations of RTOG GU Radiation Oncology Specialists Reach Consensus (11). For treatmentdesign, the Eclipse planning system was used (Varian Oncology Systems), and the isocentric intensity-modulated radiotherapy (IMRT) technique was carried out with inverse planning according to the Radiation Therapy Oncology Group (RTOG) recommendations. During therapy (image-guided radiotherapy (IGRT)), online and offline monitoring and data recording were performed by cone-beam computed tomography (CBCT).

**Figure 3:**
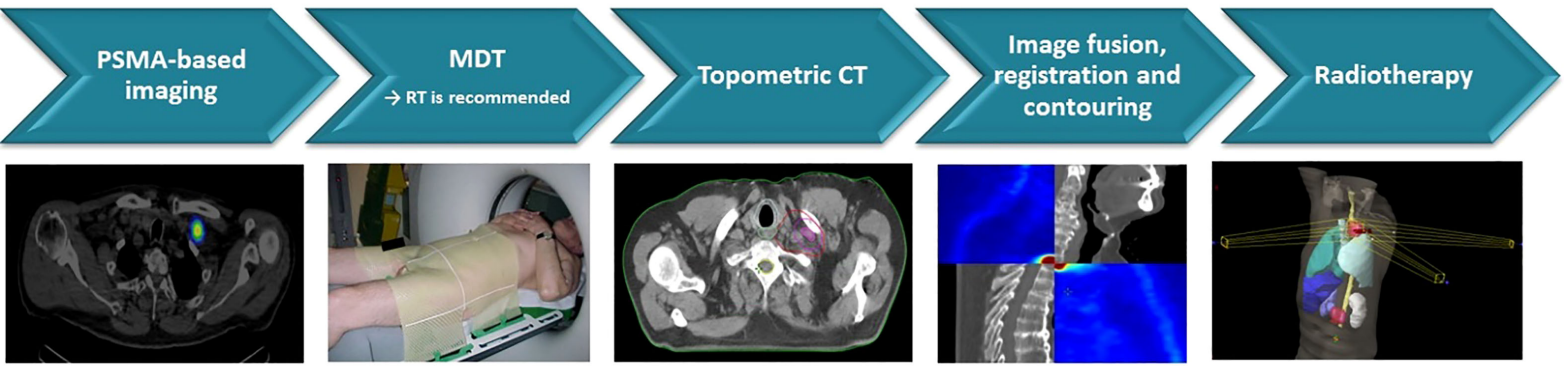
Workflow of usage of PSMA SPECT/PET/CT for GTV definition for SBRT. PSMA, prostate-specific membrane antigen; SPECT, single-photon emission computed tomography; GTV, gross tumor volume; SBRT, stereotactic body radiotherapy.

Whole-body ^99m^Tc-PSMA SPECT/CT (N = 51) or ^18^F-JK-PSMA-7 PET/CT (N = 14) was completed in some cases by a multiparametric MRI of the lesser pelvis for validation purposes. The evaluation of the scans and metabolic tumor volume contouring was performed at the Department of Nuclear Medicine, University of Szeged, whereas target volume delineation on the planning CT scan was performed at the Department of Oncotherapy, taking into account the PSMA-based imaging. Then, the comparison of the two volumes was performed by image fusion (**Figure 3**).

The PSMA isotope-based examination and the topometric CT were performed in different laying positions. While the TCT is on a straight table, the isotopic examination is on a concave one, and during the fusion of the two images, differences in size and geometry may occur. Information provided by conventional imaging was used to plan the radiation treatment, but PSMA isotope imaging was also taken into account.

### Visual analysis of PSMA imaging

The reconstructed SPECT/CT and PET/CT images were interpreted based on the reporting guidelines (12) by consensus reading of two experienced nuclear medicine and radiology specialists.

### Quantitative analysis

Tumor volume was delineated in each patient with both modalities, and gross tumor volume selection for treatment planning was also manually performed on the conventional CT-based topometric slides (GTV_CT_) by a skilled radiation oncologist. PSMA-based biological target volume (BTV_PSMA_) on PSMA-PET-CT or SPECT/CT registered images was delineated by skilled nuclear medicine and radiology specialist. GTV_CT_ and BTV_PSMA_ were delineated independently. GTV_CT_ contouring on the radiation planning CT was performed according to the guidelines of the Department of Oncotherapy based on RTOG criteria (9). Available images of former contrast CT or MRI scans were reviewed as well. Tumor volumes were determined in units of cm^3^. BTV_PSMA_ contouring was performed manually on the axial slides of ^18^F-PSMA PET or ^99m^Tc PSMA SPECT images, and the contours were finalized with the help of registered CT part of SPECT or PET images taking patient anatomy into consideration. Biological volume was also determined in cm^3^.

In each case, in each tumor target, the BTV_PSMA_ was compared to the gross tumor volume (GTV) provided by the conventional topometric CT scan, and their difference was described both in cm^3^ and in percentage.

For the topometric comparison of each tumor volume of interest (VOI), the Dice similarity coefficient (DSC) was calculated to measure the spatial overlap between the two segmentations. In the case of full overlap, the DSC is 1, whereas in full diversity, the DSC is 0 (13).

The total patient population was divided into subgroups based on applied imaging modality PSMA-PET/CT or PSMA-SPECT/CT. The aforementioned volumetric and geometrical comparisons were analyzed in both subgroups.

### Follow-up

PSA control, conventional imaging (CT, MR, and bone scintigraphy), and, in some cases, PSMA isotope-based examination were performed to determine the effectiveness of the treatment (complete remission (CR), partial remission (PR), stable disease (SD), and progressive disease (PD)).

## Results

### Patient characteristics

From 363 PSMA isotope-based examinations, 84 lesions of 64 patients were treated with stereotaxic radiation. A total of 14 exclusions occurred due to geometric distortions during image registration, mainly caused by patient motion, positioning uncertainties, and significant internal organ motions. The mean age of the patients was 66.6 (range = 55.6–79.7 years, ± SD = 6.5) years. Most of the patients being overweight the mean BMI (body mass index) was 26.96 (range=19.37-41.62 kg/m^2^) kg/m^2^. The average Gleason score was 7.9 (range = 6–10, ± SD = 1.2), and the exact breakdown of the Gleason values can be seen in **Table 1**. All patients received androgen deprivation therapy (**Table 1**). Furthermore, androgen receptor and biosynthesis inhibitor therapy (abiraterone and enzalutamide) and, in case of massive progression, docetaxel chemotherapy have been utilized. However, a detailed review of the systemic drug therapies and oncological outcomes was not in the scope of our paper.

**Table 1:** Patient characteristics.

N = 64 patients		
Mean age (years)	66.6 (55.6–79.7)	
BMI (kg/m^2^)	26.96 (19.37–41.62)	
Mean Gleason score	7.9 (6–10)	
Gleason score breakdown	5 + 5	5
	5 + 4	21
	4 + 4	11
	4 + 3	9
	3 + 4	12
	3 + 3	6
Androgen deprivation therapy	64 (100%)	
Irradiated lesion number	84	
Pre-RT mean PSA (ng/ml, ± SD)	16.98 ( ± 33.81)	
Pre-RT PSA duplication time (months, ± SD)	11.22 ( ± 38.39)	

BMI, body mass index; RT, radiation therapy; PSA, prostate-specific antigen.

### Intermodality comparison of delineated target volumes

In 50 patients, 70 lesions were examined for intermodality comparison. Of the 64 irradiated patients, 14 patients were excluded from the rigorous comparative analysis because of registration bias (patient movement, different patient position, different bladder, and intestinal status).

Tumor volumes defined by the two different imaging modalities were non-identical in 100% of the cases, decreased in size in 53 lesions (76%) of the cases, and increased in 17 lesions (24%) when the results acquired by the PSMA scan were compared to the results defined by topometric CT scan. In three out of 70 lesions, the difference in the percentage of volumes was lower than 10%. The difference in the percentage of volumes was higher than 10% in 67 out of 70 total lesions (96% of all detected lesions).

The target volume defined by the PSMA density was significantly smaller (paired t-test, p < 0.0001) than the tumor size defined by the topometric CT scan: GTV_CT_, 27.58 ± 46.07 cm^3^ (0.44–258.2 cm^3^); BTV_PSMA_, 16.14 ± 29.87 cm^3^ (0.38–190.85 cm^3^) (**Table 2**).

**Table 2:** Volume difference between GTV_CT_–BTV_PSMA_ and comparisons of subgroups.

	N	GTV_CT_ (cm^3^)	p	BTV_PSMA_ (cm^3^)	p	Volume difference (cm^3^)	p	Difference in percentage (%)	p
Volume difference between GTV_CT_ and BTV_PSMA_									
**All lesions**	**70**	27.58 ± 46.07	**-**	16.14 ± 29.87	**-**	15.04 ± 23.20 cm^3^	**-**	65.37% ± 72.70%	**-**
Comparison of subgroups defined by differences found between BTV_PSMA_ and GTV_CT_									
Group A **BTV_PSMA_> GTV_CT_**	17	8.07 ± 11.20	0.001	15.49 ± 16.03	0.918	7.41 ± 7.02	0.013	128.88 ± 122.66	0.013
Group B **BTV_PSMA_ ≤ GTV_CT_**	53	33.84 ± 51.13		16.35 ± 33.24		17.49 ± 25.96		45.00 ± 25.34	
Comparison of subgroups defined by imaging modality, differences found between BTV_PSMA_ and GTV_CT_									
Group C **PSMA-SPECT/CT**	56	32.03 ± 50.48	0.002	18.54 ± 32.76	0.181	17.40. ± 25.30	0.002	67.73 ± 79.56	0.591
Group D **PSMA-PET/CT**	14	9.77 ± 7.59		6.54 ± 8.49		5.61 ± 5.48		55.93 ± 33.90	

Group A represents patients with larger BTV_PSMA_ than GTV_CT_. Group B represents patients with BTV_PSMA_ smaller than GTV_CT_. Group C represents patients examined by PSMA-SPECT/CT. Group D represents patients examined by PSMA-PET/CT. Data are presented as mean ± standard deviation.

GTV_CT_, gross tumor volume on the topometric CT; BTV_PSMA_, biological target volume on the PSMA isotope-based examination; PSMA, prostate-specific membrane antigen; SPECT, single-photon emission computed tomography.

**Figure 4:**
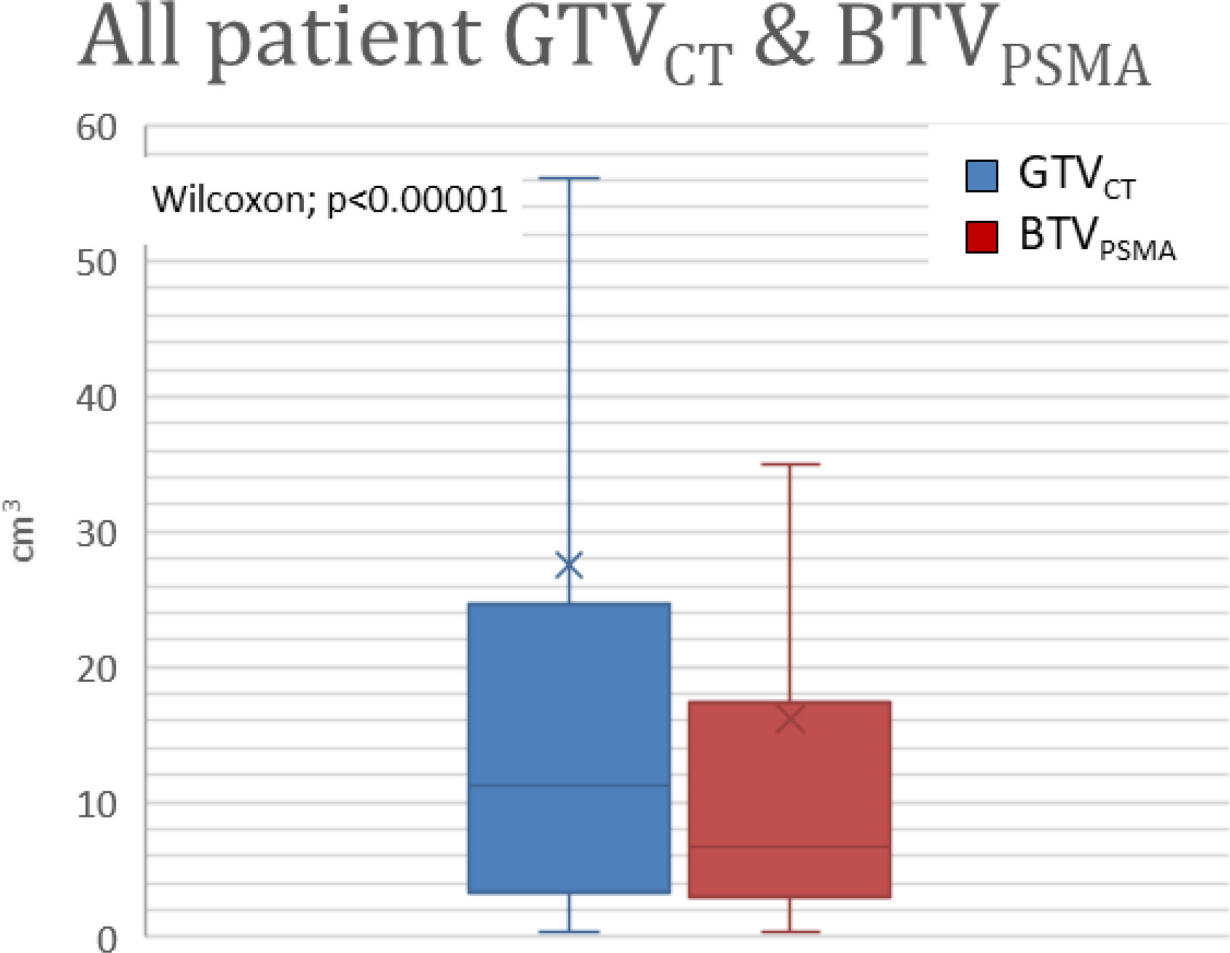
Volume difference between GTV_CT_ and BTV_PSMA_. GTV_CT_, gross tumor volume on the topometric CT; BTV_PSMA_, biological target volume on the PSMA isotope-based examination; PSMA, prostate-specific membrane antigen.

**Figure 5:**
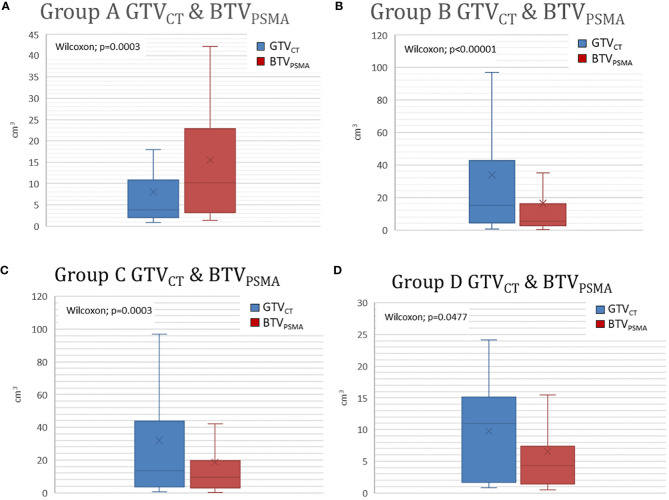
**(A, B)** Significant differences are shown in Group A and Group B between GTV_CT_ and BTV_PSMA._ Group A represents patients with larger BTV_PSMA_ than GTV_CT_. Group B represents patients with BTV_PSMA_ was smaller than GTV_CT_. **(C, D)** Modality-based subgroups showed significant differences between PSMA density-based and CT-based target volumes. Group C represents patients examined by PSMA-SPECT/CT. Group D represents patients examined by PSMA-PET/CT. GTV_CT_, gross tumor volume on the topometric CT; BTV_PSMA_, biological target volume on the PSMA-based examination; PSMA, prostate-specific membrane antigen.

Intermodality difference (independent of which modality was higher or lower) was 15.04 ± 23.20 cm^3^ (0.03–126.14 cm^3^), which describes a difference of 65.37% ± 72.70% (1.36–545.31%). During geometrical analyses, DSC was 0.56 ± 0.20 (0.07–0.85). The results of volumetric and geometrical comparisons of Group A (BTV_PSMA_ > GTV_CT_) and Group B (BTV_PSMA_ ≤ GTV_CT_) are presented (**Figures 4**, **5**).

Similar comparison data of patients acquired by SPECT/CT and PET/CT are seen in **Tables 2**, **3**.

**Table 3:** Geometry analysis, Dice similarity coefficient (DSC).

Group	N	DSC	p
**All lesions**	70	0.56 ± 0.20	–
Subgroups defined by target volume			
**Group A BTV_PSMA_ > GTV_CT_**	17	0.55 ± 0.16	0.775
**Group B** **BTV_PSMA_ ≤ GTV_CT_**	53	0.56± 0.21	
Subgroups defined by imaging modality			
**Group C** **PSMA-SPECT/CT**	56	0.55 ± 0.20	0.791
**Group D** **PSMA-PET/CT**	14	0.57 ± 0.20	

GTV_CT_, gross tumor volume on the topometric CT; BTV_PSMA_, biological target volume on the PSMA isotope-based examination; PSMA, prostate-specific membrane antigen; SPECT, single-photon emission computed tomography.

### Radiation treatment

Stereotactic treatment was performed for 84 lesions. In eight cases, local recurrence was treated; in four cases, it could only be detected by PSMA-based imaging.

In 76 lesions, metastasis-directed radiotherapy was carried out: 46 osseal, 28 lymph nodes, one adrenal gland, and one cerebral metastasis were treated. The latter was carried out in a postoperative setting.

The average gross tumor volume (GTV_CT_) was 27.58 ± 46.07 cm^3^ (0.44–258.2 cm^3^).

The prescribed doses were defined in an individualized manner, taking into consideration the localization of the tumor and eventual prior irradiation (**Table 4**).

**Table 4:** Details of radiation treatment.

Radiation therapy localization	Case	Doses (Gy)
Local recurrence	8	5 × 5.5–6 × 6
Bone	46	
- Vertebra	24	3 × 7–4 × 5–5 × 6
- Pelvic bone	12	1 × 10–5 × 6–6 × 7.5
- Rib	7	4 × 5–5 × 6–6 × 7.5
- Sternum	2	3 × 6–3 × 9
- Clavicle	1	3 × 8
Lymph node	28	3 × 8–5 × 7.25–5 × 5.5
Adrenal gland	1	5 × 6
Brain	1	3 × 9

### Follow-up

In order to evaluate the effectiveness of the treatment, PSA control was performed in every case; the mean post-RT PSA level at 3 months after SBRT was 11.19 ng/ml ( ± SD: 32.85).

**Figure 6:**
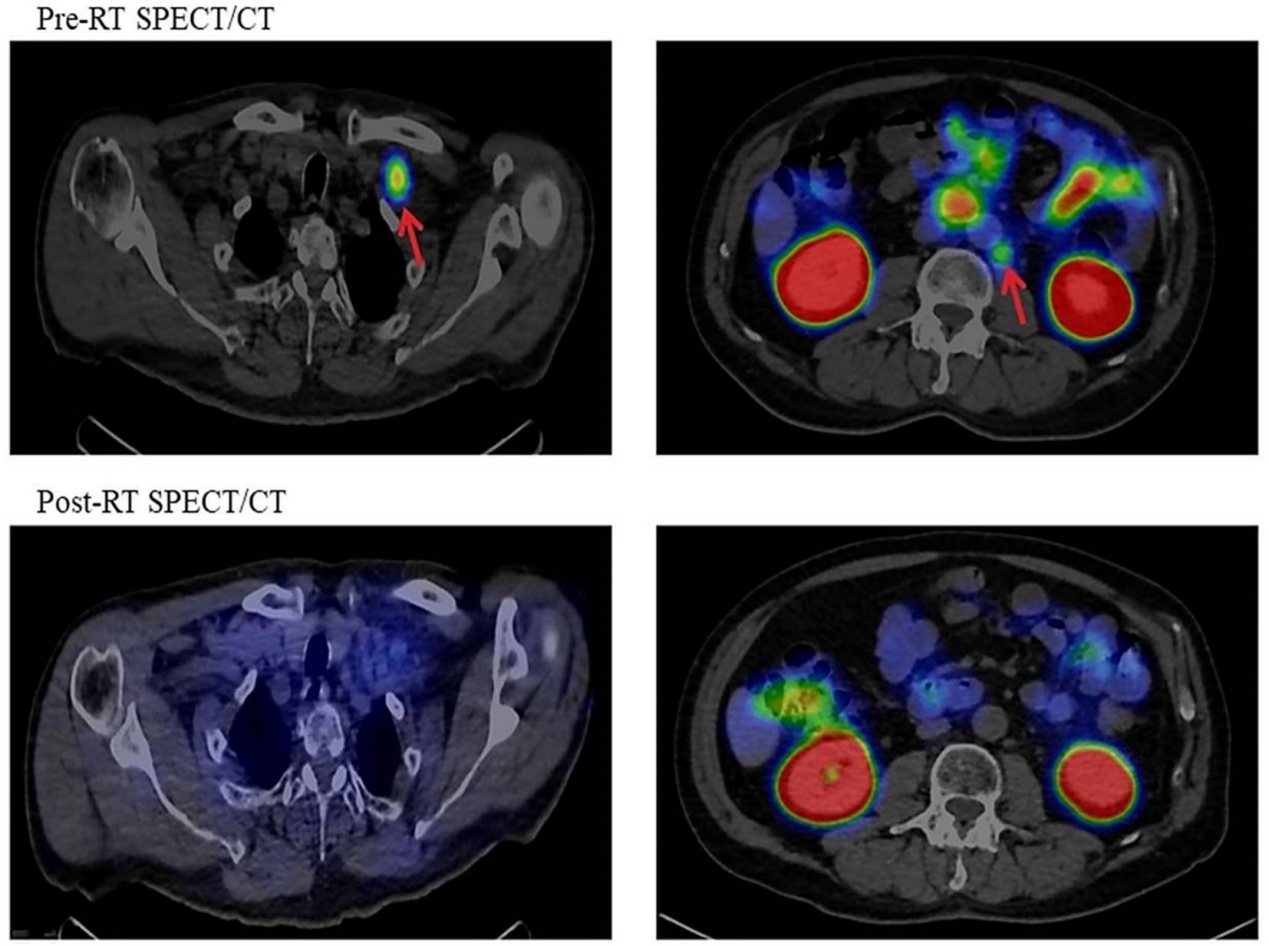
Pre- and post-RT PSMA SPECT/CT imaging. RT, radiotherapy; PSMA, prostate-specific membrane antigen; SPECT, single-photon emission computed tomography.

Three-month post-therapy PSMA-based imaging was performed in 14 cases (21.9% of the total) after radiation treatment, in which we observed a decrease or cessation of isotope uptake. Conventional imaging control was performed in 42 cases (65.6%) showing the following distribution: 22 (52.4%) CR, 14 (33.3%) PR, 4 (9.5%) SD, and 2 (4.8%) PD (**Figure 6**).

## Discussion

Biological and functional imaging approaches offer a major step to individualize radiotherapeutic treatment. The role of ^18^F-fluorodeoxyglucose (FDG)-PET for gross tumor volume identification is crucial, representing a useful and powerful tool for many tumor types, for example, pancreatic, gynecological, anal, and rectal cancers (4).

Metastasis-directed treatment (MDT) using SBRT is a new approach in the therapeutic armamentarium of oligometastatic prostate cancer, hormone-sensitive and castrate-resistant alike (14–16). The most common metastases treated with the stereotactic technique are bone and nodal lesions, but rare visceral manifestations may also be eligible for SBRT (16).

In recent years, molecular imaging techniques exerted a strong influence on the management of prostate cancer (17). These methods play a crucial role in the early diagnosis of local recurrence as well as of distant spread and can also be used in radiation therapy planning. In our article, we have exclusively focused on the latter aspect of radionuclide imaging.

Multiple radioactive tracers have been tested in prostate cancer, starting with FDG, the backbone of PET/CT imaging. Despite being the most widespread mean of tumor tracking, FDG has seen limited indications in this localization, most importantly the initial assessment of highly malignant tumors (Gleason score 7 or above) as well as the treatment response evaluation of patients with castrate-resistant metastatic disease (18).

The fine differences between SPECT and PET scans are well charted. While the spatial resolution of PET scans is somewhat higher, the functional sensibility of both methods remains high. However, for target volume definition and SBRT planning, the morphological information provided by the CT component of both SPECT/CT and PET/CT is taken into account. Therefore, the differences between the two modalities do not influence our results significantly.

In our present work, a significant number of PSMA isotope-based tests were performed on prostate carcinoma patients, of which approximately 20% were used for stereotaxic radiation treatment for postoperative local recurrence and oligometastasis. The characteristics of our patients correspond to the literature data (1). According to international guidelines, the use of androgen deprivation therapy (ADT) at advanced stages gives a survival advantage (19). In case of local recurrence, according to the recommendations of the Advanced Prostate Cancer Society, ADT is necessary (20), while in metastatic cases, continuous ADT is recommended (19).

In case of biochemical relapse after radical prostatectomy RP, which occurs in up to 50%, salvage RT of the prostate bed is performed to achieve long-term disease control depending on the stage and adverse factors (19). However, with a PSMA isotope-based test, isotope accumulation can be detected in a negative (with conventional imaging) tumor bed (21). In our present work, local recurrence was treated with SBRT in eight cases.

In a retrospective multicentric study, Kirste et al. examined the role of ^68^Ga-PSMA-PET/CT-based elective radiotherapy, evaluating the data of 394 patients with oligo-recurrent ^68^Ga-PSMA-PET/CT-positive prostate cancer. The combination of the two methods improves the outcome of oligo-recurrent prostate cancer, but there is great heterogeneity in terms of doses, treatment areas, and radiation techniques (22).

More than 50% of the metastases treated with the stereotactic radiation technique were located in the bone and approximately 40% in the lymph node, which correspond to the international distribution in the literature (19).

Although much literature data are available on SBRT for prostate cancer (PC) bone metastases (23), there are fewer clinical studies on SBRT for local recurrences and rare metastases. A systematic review carried out by Schröder et al. suggests that SBRT to the prostate bed and macroscopic recurrence remains at the experimental stage and should be utilized in distinguished cases (24). According to literature data, the treatment of adrenal metastases with stereotactic body RT provides good local control with tolerable toxicity in the case of several cancer types, including PC (25).

In one case, an asymptomatic, bifocal brain metastasis was detected by PSMA PET/CT, as a rare entity in the background of PSA progression, after metastasectomy of one of the foci histological diagnosis of prostate adenocarcinoma was proven, which is a rarity in the literature (26).

In our work, we found that GTV_CT_ contoured on the basis of conventional imaging (CT, MRI, and bone scintigraphy) appears to be larger than BTV_PSMA_ detected on the basis of PSMA isotopic examination. A close correlation between GTV_CT_ and BTV_PSMA_ was observed in 3/4 of the patients; however, in 15 patients, the volume of GTV_CT_ was significantly larger than the volume of BTV_PSMA_. In these cases, lymph node chains (three patients) or multiple vertebral metastases (12 patients) were treated. According to our current knowledge, the target volume of radiotherapy is based on conventional imaging, where radiologically enlarged lymph nodes with abnormal structure and pathological bone structure deviations need to be treated regardless of the degree of isotope accumulation.

No correlation was detected regarding the pre-RT PSA value and the pre-RT PSA duplication time and GTV_CT_ or BTV_PSMA_. Apart from increased precision, thanks to the high sensitivity of the method, recurrent disease can be detected even at very low PSA values (27), granting valuable time for treatment planning and/or inter-disciplinary consultations, which is supported by our current findings.

In a randomized two-arm clinical trial (28), 165 patients were selected for salvage radiotherapy after radical prostatectomy. In one treatment arm, only conventional imaging methods were utilized for target volume delineation, while in the other arm, additional information provided by ^18^F-fluciclovine-PET/CT was also taken into account. In 3-year event-free survival, a significant difference was observed between the two arms in favor of the latter (63% vs. 75.5%).

A retrospective study (29) demonstrated that radiotherapy plans had to be modified by an average of 60.5% due to PSMA-PET/CT findings. However, these data are to be treated with caution since the total number of patients enrolled in the trial was low (43), and the indications for irradiations were heterogeneous (curative, salvage, and metastasis treatment).

In our country, a PSMA isotope-based test can be performed in case of stage III and IV prostate cancer with an individual permit provided by the national social security.

Nowadays, PSMA PET/CT is an accepted tool in prostate cancer patient management and has become a substantial part of the imaging of PC. In some guidelines, it is the preferred method for lesion detection in biochemical relapse after primary treatment and is mandatory prior to PSMA-directed radionuclide therapy. The role of PSMA SPECT/CT is under evaluation, but SPECT/CT scan with ^99m^Tc-PSMA is also gaining acceptance to detect prostate cancer metastases. Some comparative analyses between ^68^Ga-PSMA and ^99m^Tc-PSMA have been reported. Albalooshi et al. aimed to directly compare these two techniques in patients with prostate cancer. In the 28 investigated patients, they found that in M staging, ^99m^Tc-PSMA SPECT/CT is as accurate as ^68^Ga-PSMA PET/CT, and the detection rate was not significantly different between the two techniques in patients with PSA levels >2.1 ng/ml. However, PSMA PET/CT detected more lesions (30). Fallahi et al. in patient-based evaluation showed absolute correlation between ^99m^Tc-PSMA SPECT/CT and ^68^Ga-PSMA PET/CT in 22 patients involved with metastatic lesions or highly suspicious places for the presence of metastasis (31). It is reasonable to consider using a ^99m^Tc-PSMA tracer instead of PET-based PSMA ligands. ^99m^Tc-PSMA can effectively detect metastatic lesions in prostate cancer patients with a lower financial burden and radiation exposure (32, 33).

In our recent study, the biological target volume (BTV), based on PSMA density, proved to be consequently smaller due to its functional nature, independently of whether PSMA SPECT/CT or PET/CT was performed and what the size of the lesion was. In our analysis three times more PSMA SPECT/CT could be analyzed; therefore, relevant data could be evaluated on the integration of PSMA SPECT/CT in the definition of SBRT target volume of low-volume progressing prostate cancer.

Both modalities seemed to be effective in metastatic lesion detection and target delineation; however, currently, the use of PSMA isotope-based tests is not a routine procedure in determining the radiation treatment volume.

The strength of our study is the processing of the PSMA examination of a large number of patients and the PSMA-based stereotactic radiotherapy based on the evaluation of real data. Furthermore, to the best of our knowledge, this is the first study to investigate the role of ^99m^Tc-PSMA SPECT/CT for radiotherapy planning.

A limitation of our work is that it is retrospective. The PSMA isotope-based test and the topometric CT were performed at different times and in some cases in different positions.

Clinically, we can recommend that conventional and isotopic imaging should be evaluated together, and based on these, the accurate target volume should be determined.

## Conclusions

PSMA-based imaging is a promising diagnostic method for evaluating prostate cancer. PSMA density-based functional imaging proved to be highly sensitive in detecting small lesions behind increasing PSA, which can be treated by SBRT. Its application, with the close cooperation of allied professions, facilitates the precise definition and delineation of SBRT target areas. Our results suggest that PSMA-PET or SPECT/CT can influence treatment decisions. By using conventional imaging devices (in combination with functional imaging), topometric CT (GTV_CT_), and PSMA isotopic imaging (BTV_PSMA_), more accurate target volume delineation is possible for SBRT.

In relation to the overlapping, we can assume that there is a difference in volume and geometry, and it would be advisable to use the same positioning-immobilization system.

The PSMA isotope-based tests enable a more accurate SBRT, but based on our current knowledge, the information provided by CT is also clinically necessary.

## Data availability statement

The raw data supporting the conclusions of this article will be made available by the authors, without undue reservation.

## Author contributions

LV, ZsB, and AM contributed to conception and design of the study. LV, ZsB, IF, SU, and GS organized the database. ZV performed the statistical analysis. All authors wrote sections of the manuscript. All authors contributed to the article and approved the submitted version.
